# A Comparative Study of Harmonic Scalpel Versus Electrocautery Dissection in Modified Radical Mastectomy in Breast Carcinoma Cases

**DOI:** 10.7759/cureus.66187

**Published:** 2024-08-05

**Authors:** Shriya Haval, Prabhat Nichkaode

**Affiliations:** 1 General Surgery, Dr. D. Y. Patil Vidyapeeth, Pune, IND

**Keywords:** breast carcinoma, comparative study, electrocautery, harmonic scalpel, modified radical mastectomy, operative time, postoperative pain, seroma formation, surgical outcomes

## Abstract

Aim: This study aimed to compare the outcomes of modified radical mastectomy (MRM) with the use of a harmonic scalpel versus electrocautery in patients with breast carcinoma.

Methodology: A prospective, non-randomized comparative study conducted from August 2022 to June 2024 on 40 female patients with stage II breast carcinoma undergoing MRM with electrocautery and harmonic scalpel.

Results: Patients with MRM by harmonic scalpel exhibited significantly lower intraoperative blood loss (92.50 ± 9.67 mL) than by electrocautery (172.50 ± 30.76 mL) (p-value <.0001). The average operative time was significantly shorter for the harmonic scalpel (111.00 ± 10.71 minutes) than for the electrocautery (169.50 ± 19.32 minutes) (p-value <.0001). Postoperative pain was lower for the harmonic scalpel (visual analog scale (VAS) score 3.75 ± 0.79) than for the electrocautery (VAS score 6.10 ± 0.85) (p-value <.0001). The incidence of flap necrosis was not substantially different between the categories; seroma formation was significantly lower with the use of a harmonic scalpel (p-value <.0001). Subjects in the group of harmonic scalpels also had shorter hospital stays (8.35 ± 0.93 days) compared with the electrocautery group (12.20 ± 1.06 days) (p-value <.0001).

## Introduction

In the global context, breast cancer is the most prevalent malignancy in women, representing 23% of new cases and 14% of all female fatalities [1]. The majority of cases are locally advanced illnesses; thus, modified radical mastectomy (MRM) and breast conservation surgery (BCS) remain the standard treatments [2].

A popular surgical tool for dissection and hemostasis in MRM is electrocautery, where heat is produced by passing a direct or alternating current through an electrode made of resilient metal wire, which can be applied to live tissue to either cut tissue or attain hemostasis, which has the main advantage of minimizing the loss of blood intraoperatively [3]. Previous studies have shown that electrocautery may raise the likelihood of postoperative challenges such as seroma development, wound infection, necrosis of the flap, and hematoma formation. This could lead to prolonged drainage as well as delays in the wound healing process and starting adjuvant therapy after surgery [4, 5].

The harmonic scalpel is a surgical device that utilizes ultrasonic vibrations to cut and coagulate a tissue simultaneously. It is currently often used in laparoscopic surgeries and is being explored for use in mastectomy surgeries. The blades of a harmonic scalpel vibrate at a frequency of 55,500 Hz, converting electrical energy into mechanical energy. This high-frequency vibration causes the proteins to coagulate by disrupting their hydrogen bonds. As a result, tissues get cut and coagulated, and vascular and lymphatic capillaries are sealed. This reduces tissue injury by causing less heat dispersion than monopolar electrocautery [6, 7].

This study aimed to compare the outcome factors in MRM using a harmonic scalpel versus electrocautery intraoperatively and postoperatively in terms of intraoperative volume of blood loss, operative time, postoperative pain, formation of seroma and flap necrosis, and recovery.

## Materials and methods

This prospective, non-randomized comparative study was conducted at a tertiary hospital from August 2022 to June 2024. Ethical approval for this study was obtained from the Institutional Review Board Ethics Committee, Dr. D Y Patil Vidyapeeth, Pune (approval number: IESC/PGS/2022/78). All participants provided written informed consent prior to enrolment in the study. The consent form included information about the study's purpose, procedures, potential risks, and benefits. Participants were assured of the confidentiality of their data and their right to withdraw from the study at any time without any impact on their medical care.

The mean and standard deviation of drain output in Group A (ultrasonic dissector) and Group B (electrocautery) were considered as 61.00 (40.38) and 161.00 (40.38), respectively, in the study by Deori et al. [8]. The study was conducted with a 95% confidence interval, 80% power, and 10% loss due to follow-up. The sample size was calculated as 40 with 20 in each group. The software used is WinPepi version 11.65 (http://www.brixtonhealth.com/pepi4windows.html).

A total of 66 patients with breast lumps were initially admitted, and a sample of 40 patients aged between 18 and 80 years was selected. These subjects had Stage II breast carcinoma and palpable ipsilateral axillary lymph nodes, with no indication of BCS. These patients also had biopsy-proven infiltrating ductal carcinoma of the breast and no evidence of distant metastasis on contrast-enhanced CT of the chest, abdomen, and pelvis. The remaining 26 patients were excluded due to factors such as breast carcinoma in pregnancy, male patients with breast carcinoma, locally advanced carcinoma of the breast, recurrent carcinoma of the breast, prior chest wall radiotherapy for breast carcinoma, triple-negative receptor status (estrogen receptor (ER) positive, progesterone receptor (PR) positive, human epidermal growth factor receptor 2 (HER2)/neu), presence of any comorbidities, hemodynamic instability, or unwillingness to participate.

**Figure 1:**
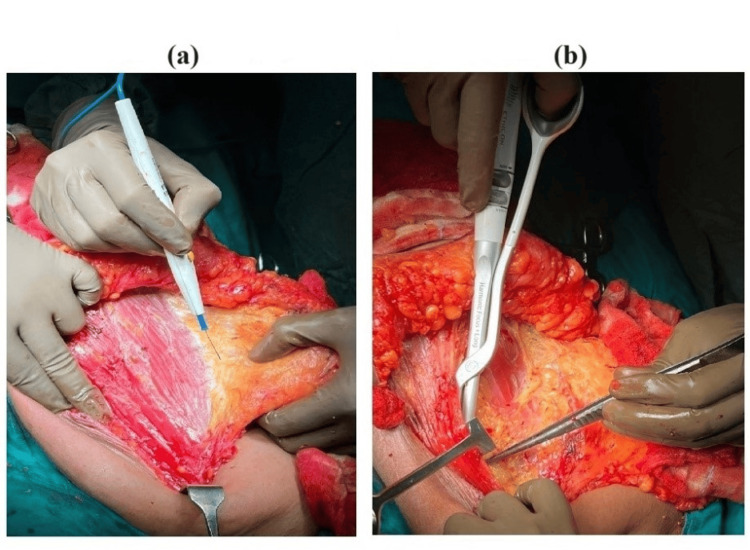
Intraoperative images showing sources of energy used in MRM (a) Electrocautery being used for raising flaps in MRM; (b) harmonic scalpel being used for axillary dissection in MRM MRM: modified radical mastectomy

**Figure 2:**
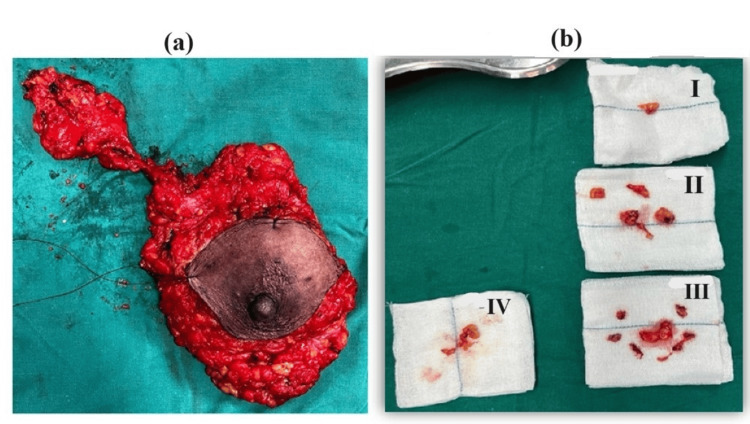
Excised specimens after MRM (a) Entire right breast with lump and axillary tail specimen; (b) I, II, III, IV-Level I, II LN, apical node, and Rotter's node, respectively, sampled from the ipsilateral axilla MRM: modified radical mastectomy; LN: lymph node

All 40 study participants, after careful perioperative evaluation, fluid management, and fitness from the anesthetist, underwent a standard surgical technique of Auchincloss-modified radical mastectomy under general anesthesia. Surgery was performed by a surgeon at the professor level with experience of 250 MRMs. Of these 40 subjects, 20 had their dissection performed using electrocautery (Figure 1a) and were designated as Group A. The remaining 20 patients underwent the dissection process with a harmonic scalpel (Figure 1b) and comprised Group B. The excised specimens (Figures 2a-2b) were sent for histopathological examination.

The following post-MRM outcome factors were evaluated: volume of blood loss during surgery, operating time (time from the first incision to last suture), postoperative pain (by visual analog scale (VAS) score), flap necrosis, seroma formation (based on total drain output and duration of drain kept in situ post surgery), and the time between undergoing surgery and returning to work.

Statistical analysis

Data were gathered and graphs were created using Microsoft Excel 2019 (Microsoft Corp., Redmond, WA). The data were evaluated using IBM SPSS Statistics software for Windows, version 23.0 (IBM Corp., Armonk, NY). Before performing the statistical tests, the normality of the data was assessed using the Shapiro-Wilk test. This test was chosen due to its suitability for small sample sizes (n <50). For continuous variables, data were represented by mean (standard deviation, SD). The independent sample t-test was used to compare the means between Group A (electrocautery) and Group B (harmonic scalpel). Pearson's chi-square test was used to evaluate the relationship between categorical variables.

The results were interpreted based on the p-values obtained from the statistical tests. A p-value less than 0.05 was considered indicative of a statistically significant difference between the two groups.

## Results

We compared two different energy sources for dissection during MRM. Group A underwent MRM with electrocautery, while Group B underwent MRM with the harmonic scalpel. The majority of patients, 14 out of 40 patients (35%), were aged between 41 and 50 years.

A substantially considerable difference (p-value <.0001) in the mean postoperative VAS score between Group A (6.10 ± 0.85) and Group B (3.75 ± 0.79) was noted (Table 1).

**Table 1:** Comparison of outcomes in MRM across both groups MRM: modified radical mastectomy An independent sample t-test was employed to evaluate continuous variables, and a two-tailed p-value less than 0.05 was considered statistically significant.

S. No.	Outcomes evaluated	Group A: Electrocautery (total = 20) mean ± standard deviation	Group B: Harmonic scalpel (total = 20) mean ± standard deviation	p-value
1.	Volume of blood loss	172.50 ± 30.76 milliliter	92.50 ± 9.67 milliliter	< .0001
2.	Duration of surgery	169.50 ± 19.32 minutes	111.00 ± 10.71 minutes	< .0001
3.	Postoperative visual analog scale score (from 1 to 10)	6.10 ± 0.85	3.75 ± 0.79	< .0001
5.	Duration of drain	9.80 ± 1.01 days	6.80 ± 0.83 days	<0.0001
6.	Total drain output	894.50 ± 90.58 milliliter	475.00 ± 65.97 milliliter	< .0001
7.	Hospital stay	12.20 ± 1.06 days	8.35 ± 0.93 days	< .0001

Until postoperative day (POD) four, three patients (15%) in Group A and seven patients (35%) in Group B had flap necrosis. Pearson's chi-square test results revealed that this was not a statistically significant difference (p-value = .144)

Also, Group B had an average duration for which the drain was kept in situ of 6.80 ± 0.83 days, whereas Group A had an average of 9.80 ± 1.01 days. This difference was statistically significant (p-value <.0001). (Table 1). Another statistically significant result (p-value <.0001) was observed between Group B (475.00 ± 65.97 mL) and Group A (894.50 ± 90.58 mL) in terms of average total drain output (Table 1).

The final comparison showed that Group A had a mean hospital stay of 12.20 ± 1.06 days, whereas Group B had a mean hospital stay of 8.35 ± 0.93 days. This difference was statistically significant (p-value <.0001) (Table 1).

## Discussion

The subgroup analysis revealed that the average blood loss was more for electrocautery (Group A), whereas the average blood loss for the harmonic scalpel (Group B) was significantly less, which, when compared to previous studies by Mittal et al. [9], Khan et al. [10], Adwani and Ebbs [11], Archana et al. [12], and Chejara et al. [13], which supported this result. Therefore, we inferred that in MRM, using a harmonic scale significantly lowers intraoperative blood loss.

While analyzing the operative duration in both groups, it showed that less operative time was needed with a harmonic scalpel, and previous research done by Archana et al. [12], Galatius et al. [14], and Ribeiro et al. [15] corroborated this finding. However, in a study by Mittal et al. [9], using a harmonic scalpel significantly increased the mean operating time. This discrepancy in the results may be related to a lack of experience and orientation to use a harmonic scalpel in comparison to standard electrocautery [9].

The subgroup analysis of postoperative pain showed that the harmonic scalpel (Group B) had a significantly lower VAS score than that of electrocautery (Group A). These findings align with prior research conducted by Khan et al. [10], Archana et al. [12], and Chejara et al. [13]. This is due to the limited lateral thermal damage caused by the harmonic scalpel, which causes a reduction in the activation of pain nerve endings [7]. Thus, using a harmonic scalpel appears to significantly reduce postoperative discomfort for patients.

We also found that there was no significant difference in the development of flap necrosis across both groups, which was consistent with research conducted by Mittal et al. [9], Archana et al. [12], and Chejara et al. [13]. Thus, we can state that neither of the MRM techniques affects the development of flap necrosis.

For analyzing the formation of postoperative seroma in both groups, we studied the total drain volume and the duration of drain kept in situ. The total drain output was higher in patients who underwent MRM with electrocautery (Group A) than in Group B. These results were similar to data analyzed and reported by Mittal et al. [9], Khan et al. [10], Muhammad et al. [16], and Deo et al. [17]. Additionally, the duration of drain kept in situ for Group A was more and was less in Group B. Similar results were observed in studies conducted by Khan et al. [10], Muhammad et al. [16], Deo et al. [17], and Faisal et al. [18]. Thus, it appears that the use of a harmonic scalpel results in significantly less seroma formation than electrocautery.

According to our findings, patients in Group A had a longer post-surgery hospital stay than Group B. On regular follow-up, we observed that within three weeks of being discharged, patients in Group B were able to resume their regular activities without any issues. This finding is supported by previous studies by Muhammad et al. [16] and Cheng et al. [19]. In other words, patients who had MRM using a harmonic scalpel recovered faster and could go back to their regular activities earlier than patients who had MRM using electrocautery.

Study limitations

The study, which involved only 40 participants, has a relatively small sample size, limiting the robustness and generalizability of its results. Conducting the research at a single tertiary hospital further constrains the applicability of the findings, as multi-center studies would be necessary to validate the results across different settings and populations.

The study was performed by a highly experienced surgeon who had completed 250 MRMs, raising concerns about whether the outcomes can be replicated in settings with less experienced surgeons. The surgical technique itself can vary between surgeons and institutions, affecting the consistency of the results. Surgeons’ experience and familiarity with the harmonic scalpel or electrocautery can influence the efficiency and effectiveness of the procedure.

The study excluded patients with comorbidities such as diabetes, hypertension, and cardiovascular diseases that can impact wound healing, pain perception, and overall recovery time. Perioperative hemodynamics, including blood pressure control and fluid management, also play a critical role in surgical outcomes. Fluctuations in blood pressure and poor perioperative management can increase the risk of complications such as bleeding and flap necrosis. Patients with such comorbidities may experience different outcomes compared to healthier individuals.

Additionally, the study does not address the cost implications comprehensively. While the harmonic scalpel is more expensive, it is associated with fewer complications, potentially resulting in lower overall healthcare costs. Another limitation is the reusability of the harmonic scalpel, which can only be sterilized and reused five to six times, potentially increasing costs and logistical challenges.

Also, as the specific focus was on patients with stage II breast carcinoma, the findings may not apply to patients with other stages of breast cancer. More research is needed to confirm these results in a broader range of disease stages.

Finally, the study's follow-up period was relatively short, limited to the time it took patients to resume their daily activities (four to six weeks). Consequently, the incidence of lymphedema formation, a significant post-surgical complication, was not assessed, necessitating longer-term follow-up studies to capture these outcomes.

## Conclusions

We conclude that the use of the harmonic scalpel in MRM for breast carcinoma significantly improves intraoperative and postoperative outcomes compared to electrocautery. This could shift current surgical practices by highlighting the harmonic scalpel's advantages, such as reduced blood loss, shorter operative time, less postoperative pain, and quicker recovery. These benefits support the adoption of a harmonic scalpel as a viable alternative to electrocautery and could lead to widespread adoption of the harmonic scalpel in breast carcinoma surgeries, ultimately enhancing patient care and surgical efficiency.

## References

[1] Jemal A, Bray F, Center MM, Ferlay J, Ward E, Forman D (2011). Global cancer statistics. CA Cancer J Clin.

[2] Fitzmaurice C, Abate D, Abbasi N (2019). Global, regional, and national cancer incidence, mortality, years of life lost, years lived with disability, and disability-adjusted life-years for 29 cancer groups, 1990 to 2017: a systematic analysis for the Global Burden of Disease study. JAMA Oncol.

[3] Sheen-Chen SM, Chou FF (1993). A comparison between scalpel and electrocautery in modified radical mastectomy. Eur J Surg.

[4] Porter KA, O'Connor S, Rimm E, Lopez M (1998). Electrocautery as a factor in seroma formation following mastectomy. Am J Surg.

[5] Deo SV, Shukla NK (2000). Modified radical mastectomy using harmonic scalpel. J Surg Oncol.

[6] Hambley R, Hebda PA, Abell E, Cohen BA, Jegasothy BV (1988). Wound healing of skin incisions produced by ultrasonically vibrating knife, scalpel, electrosurgery, and carbon dioxide laser. J Dermatol Surg Oncol.

[7] Hoenig DM, Chrostek CA, Amaral JF (1996). Laparosonic coagulating shears: alternative method of hemostatic control of unsupported tissue. J Endourol.

[8] Deori A, Gupta N, Gupta AK, Yelamanchi R, Agrawal H, Durga CK (2021). A prospective randomised controlled study comparing ultrasonic dissector with electrocautery for axillary dissection in patients of carcinoma breast. Malays J Med Sci.

[9] Mittal P, Kumar A, Kaur S, Pandove PK, Singla RL, Singh J (2017). A comparative study of the use of harmonic scalpel versus unipolar cautery in modified radical mastectomy. Niger J Surg.

[10] Khan S, Khan S, Chawla T, Murtaza G (2014). Harmonic scalpel versus electrocautery dissection in modified radical mastectomy: a randomized controlled trial. Ann Surg Oncol.

[11] Adwani A, Ebbs SR (2006). Ultracision reduces acute blood loss but not seroma formation after mastectomy and axillary dissection: a pilot study. Int J Clin Pract.

[12] Archana A, Sureshkumar S, Vijayakumar C, Palanivel C (2018). Comparing the harmonic scalpel with electrocautery in reducing postoperative flap necrosis and seroma formation after modified radical mastectomy in carcinoma breast patients: a double-blind prospective randomized control trial. Cureus.

[13] Chejara R, Ranjithkumar M, Arya S (2020). A comparative study of harmonic scalpel versus electrocautery dissection in modified radical mastectomy. SAS J Surg.

[14] Galatius H, Okholm M, Hoffmann J (2003). Mastectomy using ultrasonic dissection: effect on seroma formation. Breast.

[15] Ribeiro GH, Kerr LM, Haikel RL (2013). Modified radical mastectomy: a pilot clinical trial comparing the use of conventional electric scalpel and harmonic scalpel. Int J Surg.

[16] Muhammad R, Johann KF, Saladina JJ, Harlina ML, Niza AS (2013). Ultracision versus electrocautery in performing modified radical mastectomy and axillary lymph node dissection for breast cancer: a prospective randomized controlled trial. Med J Malaysia.

[17] Deo SV, Shukla NK, Asthana S, Niranjan B, Srinivas G (2002). A comparative study of modified radical mastectomy using harmonic scalpel and electrocautery. Singapore Med J.

[18] Faisal M, Fathy H, Shaban H, Abuelela ST, Marie A, Khaled I (2018). A novel technique of harmonic tissue dissection reduces seroma formation after modified radical mastectomy compared to conventional electrocautery: a single-blind randomized controlled trial. Patient Saf Surg.

[19] Cheng H, Clymer JW, Ferko NC, Patel L, Soleas IM, Cameron CG, Hinoul P (2016). A systematic review and meta-analysis of harmonic technology compared with conventional techniques in mastectomy and breast-conserving surgery with lymphadenectomy for breast cancer. Breast Cancer (Dove Med Press).

